# Hepatic venous pressure gradient and rebleeding risk of patients with nonalcoholic steatohepatitis cirrhosis after variceal bleeding

**DOI:** 10.3389/fmed.2023.1224506

**Published:** 2023-07-25

**Authors:** Yiqi Shi, Wenyong Shen, Gang Xu, Xunzheng Wang, Bo Ning

**Affiliations:** ^1^Digestive System Department, Yuzhong Hospital of the Second Affiliated Hospital of Chongqing Medical University, Chongqing, China; ^2^Digestive System Department, Chongqing Fuling Central Hospital of Chongqing University, Chongqing, China; ^3^Digestive System Department, Jiangnan Hospital of the Second Affiliated Hospital of Chongqing Medical University, Chongqing, China

**Keywords:** nonalcoholic steatohepatitis (NASH), hepatitis B virus (HBV), transjugular intrahepatic portosystemic shunt (TIPS), variceal bleeding, hepatic venous pressure gradient (HVPG)

## Abstract

**Background and aims:**

Hepatic venous pressure gradient (HVPG) has a strong predictive value for variceal rebleeding in cirrhotic patients, but the accuracy of HVPG may be compromised in nonalcoholic steatohepatitis (NASH) cirrhosis. This study aimed to evaluate the accuracy of HVPG and portal pressure gradient (PPG) for predicting rebleeding in NASH cirrhosis after acute variceal bleeding.

**Patients and methods:**

Thirty-eight NASH cirrhosis patients and 82 hepatitis B virus (HBV) cirrhosis patients with acute variceal bleeding were included in this study. All patients recived transjugular intrahepatic portalsystemic shunt (TIPS). The prognostic value of HVPG and PPG for variceal rebleeding was evaluated.

**Results:**

Compared with HBV cirrhosis, NASH cirrhosis demonstrated a lower HVPG (15.3 ± 3.8 vs. 18.0 ± 4.8; *p* = 0.003) and lower PPG (18.0 ± 3.7 vs. 20.0 ± 3.4; *p* = 0.005). HVPG (AUC = 0.82; *p =* 0.002) and PPG (AUC = 0.72; *p* = 0.027) had promising prognostic value among NASH cirrhosis patients. The optimal threshold of HVPG and PPG for predicting rebleeding in NASH cirrhosis was 17 mmHg and 20 mmHg. At multivariate analysis, HVPG ≥17 mmHg was a significant predictor of variceal rebleeding (HR 9.40; 95% CI 1.85–47.70; *p =* 0.007).

**Conclusion:**

In the patients with cirrhosis and vairceal bleeding, the levels of HVPG and PPG were found to be low in NASH cirrhosis than HBV cirrhosis. However, the prevalence of rebleeding was similar between two groups. HVPG measurement is still an accurate way to assess the risk of variceal rebleeding in NASH cirrhosis.

## Introduction

Nonalcoholic fatty liver disease (NAFLD), also known as metabolic dysfunction-associated fatty liver disease, has become an important public health concern and has a global prevalence of 25% (1). Nonalcoholic steatohepatitis (NASH) (a severe form of NAFLD typically characterized by lobular inflammation, ballooning degeneration and fibrosis) can progress to end-stage liver disease, such as cirrhosis and hepatocellular carcinoma (HCC), and eventually liver-related mortality (2–4). Approximately 20% of patients with NASH will progress to cirrhosis and encounter cirrhosis-associated decompensation outcomes (e.g., variceal bleeding, hepatic encephalopathy, hepatorenal syndrome, and ascites) (5, 6). Therefore, early identification of patients at high risk for cirrhosis-related complications is beneficial for prognosis in NASH cirrhosis.

Hepatic venous pressure gradient (HVPG) is considered the surrogate marker of portal pressure gradient (PPG) and represents standard reference for staging cirrhotic portal hypertensio (7–9). HVPG value >5 mmHg indicates portal hypertension, and a value >10 mmHg indicates clinically significant portal hypertension (CSPH), while HVPG value of 20 mmHg or higher predicts a high incidence of acute variceal hemorrhage at endoscopy and a high mortality (10, 11). Several studies have evaluated the capacity of HVPG to correspond to liver-related complications, especially in viral and alcoholic cirrhosis, but few studies have focused on HVPG measurement in clinically decompensated NASH cirrhosis and its correlation with variceal rebleeding. The predictive value of HVPG in previous investigations was controversial, and NASH patients had similar portal hypertensive complications at lower HVPG compared with other liver disease etiologies (7, 12). Under normal conditions, HVPG greater than 10 mmHg predisposes patients to esophageal variceal bleeding and other portal hypertension-related complications. However, HVPG of no more than 10 mmHg in NASH may lead to the above-described complications (13, 14). On the other hand, a reduction in HVPG in each stage of NASH fibrosis was observed compared to hepatitis C virus (HCV) disease (15), which raises the concern of whether HVPG is accurate in predicting evaluating portal hypertensive complications in NASH cirrhosis.

Variceal bleeding is a life-threatening complication with a high rebleeding rate and mortality among portal hypertension-related events. Even if variceal bleeding is controlled, stricter means are needed to monitor and prevent rebleeding. Within the first days following an initial hemorrhage episode, the mortality rate reaches 20%, and the rebleeding rate is as high as 30–50% (16, 17). Therefore, we applied this study to compare portal and hepatic venous pressure among patients with NASH cirrhosis and HBV cirrhosis and to evaluate the accuracy of HVPG for predicting variceal rebleeding and other clinical decompensation events.

## Patients and methods

### Patients

Forty-six NASH cirrhosis patients and 146 HBV cirrhosis patients underwent transjugular intrahepatic portosystemic shunt (TIPS) due to acute variceal bleeding in three tertiary medical centers (Yuzhong Hospital of the Second Affiliated Hospital of Chongqing Medical University, Jiangnan Hospital of the Second Affiliated Hospital of Chongqing Medical University and Chongqing Fuling Central Hospital of Chongqing University) from February 2017 to March 2021 were enrolled. All patients were followed up until September 2021. All patients signed informed consent forms. The research was approved by the Ethics Committee of the Second Affiliated Hospital of Chongqing Medical University. NASH cirrhosis was diagnosed in patients with fatty liver who developed cirrhotic signs confirmed *via* histological and imaging evidence and at least one metabolic risk factor without a history of alcohol abuse and other known causes of chronic liver disease. The metabolic risk factors included being overweight or obese (body mass index (BMI) ≥25 kg/m^2^), hypertension, diabetes mellitus and hyperlipidemia. All HBV patients had evidence of HBV infection (HBV surface antigen and HBV DNA positive).

The inclusion criteria were as follows: (1) decompensated NASH cirrhosis or HBV cirrhosis (histological or/and radiological criteria), (2) clinical manifestations of hematemesis and/or melena, (3) acute variceal bleeding confirmed by endoscopy according to Baveno II criteria (18), (4) age > 18 years and < 80 years; (5) absence of liver transplantation, and (6) no significant alcohol abuse.

The exclusion criteria were as follows: (1) advanced hepatocellular carcinoma according to Milan criteria, (2) absence of hemodynamic measurement, (3) previous treatment of portal hypertension and its complications, such as TIPS placement and endoscopic treatment for variceal bleeding, (4) Child–Pugh score > 13, (5) complete portal vein thrombosis, (6) bleeding from ectopic varices, and (7) comorbidities and medications that may affect portal hypertension and gastrointestinal bleeding, such as heart failure, peptic ulcer, beta-blocker, and antithrombotic therapy.

### Interventions

After admission, clinical history, physical examination, laboratory tests, and radiological imaging (hepatic portal vein computed tomography angiography) were performed. All patients admitted for variceal bleeding were first treated with proton pump inhibitors and vasoactive drugs (terlipressin and octreotide). Blood and glucose-electrolyte solutions were transfused to maintain hemodynamic stability. They received early endoscopic treatment within 24 h after admission. Endoscopic treatment for esophageal and gastric varices included endoscopic variceal ligation (multiband ligation device [Wilson-Cook Medical]) and histoacryl injection. TIPS placement was performed, and portal hypertension was evaluated during the first 48 h after bleeding when patients were under a stable hemodynamic condition.

### TIPS placement

The measurement of HVPG was performed during the TIPS procedure and adherence to standard operating procedures. Strict quality control standards were established to ensure the reliability of pressure measurement during the procedures. All procedures were performed under conscious sedation and local anesthesia. Using the transjugular approach, a transjugular liver access set (Cook, Bloomington, IN, United States) was guided into the inferior vena cave, right hepatic vein and portal vein. Viatorr^®^ PTFE-covered stents (Gore, Flagstaff, AZ, United States) were implanted following balloon dilation. Embolization of the gastric coronary vein was considered when portography clearly showed dilatation of the gastric coronary vein. The preoperative HVPG, preoperative PPG and the postoperative PPG were measured. The HVPG was obtained by calculating the difference between the wedged hepatic venous pressure (WHVP) and the free hepatic venous pressure (FHVP). The PPG was obtained by calculating the difference between the portal pressure (PP) and the inferior vena cava pressure.

### Outcomes and follow-up

The primary endpoint was variceal rebleeding, defined as hematemesis and/or melena according to the Baveno Consensus (19). Variceal rebleeding was diagnosed using endoscopy when varices were bleeding, or signs of recent bleeding were observed, and varices were the only potential source of bleeding. The secondary endpoints were: shunt dysfunction defined as a maximum intrastent flow velocity less than 50 cm/s or higher than 200 cm/s, hepatic encephalopathy, new or recurrent ascites, liver failure, hepatocellular carcinoma and overall survival.

Patients were followed up using endoscopy, biochemical assessment and Doppler ultrasonography every 1, 3 and 6 months after TIPS and every year thereafter. Shunt patency and blood flow velocity was assessed by Doppler Ultrasound. Survival was calculated from the date after surgery to mortality or the latest follow-up. Patients were encouraged to quit smoking and alcohol and maintain a low-fat and low-carbohydrate diet during the follow-ups.

### Statistical analysis

SPSS version 26.0 was used for statistical analysis. Continuous variables are expressed as the mean and standard deviation, and an unpaired Student’s t test or the Mann–Whitney test was used to compare groups. Count variables are expressed as constituent ratios or rates, and Pearson’s χ^2^ or Fisher’s exact test was used to comparing groups. Correlation was calculated by Pearson correlation. Cumulative probabilities of clinical outcomes were analyzed using the competing risk model. Survival was assessed by Kaplan–Meier and log-rank test. Both univariate and multivariate analyzes were used to assess the risk factors associated with variceal rebleeding using the Cox proportional hazard regression model. Discrimination of predictive variables for rebleeding was performed using logistic regression models. Moreover, we identified the optimal cutoff values using logistic regression by calculating the area under the receiver operating characteristic curve (AUC). *p* ≤ 0.05 was considered statistically significant.

## Results

### Baseline characteristics of patients

Between February 2017 and March 2021, 192 patients with NASH cirrhosis or HBV cirrhosis with acute variceal bleeding were enrolled. A total of 38 patients were excluded due to incomplete information, 10 due to portal vein thrombosis, 10 due to previous TIPS treatment, 8 due to hepatocellular carcinoma, 4 due to obviously impaired liver function with a Child–Pugh score > 13, and 2 due to ectopic variceal bleeding. Among the 120 included patients, 38 (31.7%) had NASH cirrhosis, and 82 (68.3%) had HBV cirrhosis.

The baseline characteristics of all patients with NASH cirrhosis and HBV cirrhosis are shown in Table 1. The mean age of NASH cirrhosis was 56.7 (interquartile range [IQR], 50–65) years, the mean follow-up time was 27.6 months, and that of HBV cirrhosis was 49.2 years (IQR 44–56) and 24.7 months, respectively. Among patients with NASH cirrhosis, the proportion of females was 55.3%, which was significantly higher than that of patients with HBV cirrhosis (17%, *p* < 0.001). Nineteen patients (50%) with NASH cirrhosis had metabolic syndrome, 18 patients (47.4%) were overweight or obese, 28 patients (73.7%) had diabetes mellitus, 10 patients (26.3%) had hypertriglyceridemia, and 8 patients (21%) had hypertension.

**Table 1:** Baseline demographics and characteristics of patients.

	NASH (n = 38)	HBV (n = 82)	*p* value
Age	56.7 ± 8.8	49.2 ± 9.4	<0.001
Female	21 (55.3)	17 (20.7)	<0.001
BMI (kg/m^2^)	24.7 ± 4.1	21.7 ± 2.6	0.032
Overweight/Obese	18 (47.4)	9 (11.0)	<0.001
Ascites			0.319
Mild	15 (39.5)	33 (40.2)	
Moderate/Excessive	4 (10.5)	17 (20.7)	
Metabolic syndrome	19 (50.0)	6 (7.3)	<0.001
Esophageal varices	34 (89.5)	77 (93.9)	0.392
Gastric varices	30 (78.9)	66 (80.5)	0.844
Hypertension	8 (21.0)	5 (6.1)	0.014
Diabetes	28 (73.7)	27 (32.9)	<0.001
Hypertriglyceridemia	10 (26.3)	0	<0.001
Child–Pugh score	6.7 ± 1.5	7.2 ± 1.7	0.097
Child–Pugh class			0.124
Child class A	22 (57.9)	33 (40.2)	
Child class B	15 (39.5)	40 (48.9)	
Child class C	1 (2.6)	9 (11.0)	
MELD	10.5 ± 2.4	11.8 ± 3.2	0.034
Platelets (×10^9^/L)	83.5 ± 41.2	67.0 ± 49.7	0.003
Albumin (g/dL)	3.4 ± 0.6	3.3 ± 0.6	0.520
Bilirubin (mg/dL)	1.4 ± 0.9	1.6 ± 1.3	0.254
ALT (U/L)	27.4 ± 17.8	42.3 ± 55.8	0.031
AST (U/L)	38.1 ± 27.9	42.5 ± 41.1	0.492
GGT (U/L)	64.2 ± 71.4	43.4 ± 35.4	0.034
AP (U/L)	92.7 ± 56.6	80.5 ± 28.3	0.118
INR	1.3 ± 0.2	1.4 ± 0.3	0.004
Serum creatinine (mg/dL)	0.8 ± 0.2	0.7 ± 0.2	0.602
Serum sodium (mmol/L)	138.9 ± 3.6	138.0 ± 4.3	0.247
Portal vein diameter (mm)	15.6 ± 3.1	16.8 ± 3.3	0.030

BMI, body mass index; HCC, hepatocellular carcinoma; MELD, model of end-stage liver disease score; ALT, alanine aminotransferase; AST, aspartate aminotransferase; GGT, gamma-glutamyl transpeptidase; AP, alkaline phosphatase; INR, international normalized ratio.

Biochemical analysis of liver function showed that NASH cirrhosis patients had better liver function results and significantly lower Model for End-Stage Liver Disease (MELD) scores (*p* = 0.034). Alanine aminotransferase (*p* = 0.031) and the international normalized ratio (*p* = 0.004) were significantly higher in the HBV group, while the level of gamma-glutamyltransferase (GGT) was significantly higher in the NASH group (*p* = 0.034). In addition, the platelet count was higher in NASH cirrhosis (*p* = 0.003).

### HVPG/PPG measurement

Patients with NASH cirrhosis had a lower portal pressure (26.3 ± 6.1 vs. 30.1 ± 4.7; *p* < 0.001), lower WHVP (24.1 ± 5.3 vs. 27.6 ± 5.5; *p* = 0.001), lower HVPG (15.3 ± 3.8 vs. 18.0 ± 4.8; *p* = 0.003) and lower PPG (18.0 ± 3.7 vs. 20.0 ± 3.4; *p* = 0.007) than those with HBV cirrhosis (Table 2). Remarkable correlation between the HVPG and PPG was determined by a Pearson correlation coefficient of 0.78. The R-squared value was 0.608. High HVPG levels were more frequently found in HBV cirrhosis. The HVPG level in 3 (7.9%) NASH patients versus 25 (30.5%) HBV patients was greater than or equal to 20 mmHg (*p* = 0.006). Low HVPG (<10 mmHg) levels were observed in 3 (7.9%) NASH patients and 3 (3.7%) HBV patients. After successful TIPS treatment, the PPG significantly decreased from 18.0 ± 3.7 mmHg vs. 20.0 ± 3.4 mmHg to 7.6 ± 4.1 mmHg vs. 9.2 ± 3.8 mmHg (NASH cirrhosis vs. HBV cirrhosis,). The PPG level after TIPS treatment of NASH cirrhosis was significantly lower than that after TIPS treatment of HBV cirrhosis (*p* = 0.04). Compared to the baseline, an PPG value, a mean reduction of 10.4 mmHg was observed in NASH cirrhosis and 10.8 mmHg in HBV cirrhosis. No significant difference was found between them. After TIPS treatment, the PPG effectively decreased to a level of <12 mmHg in 104 (86.7%) patients, but all patients achieved a sufficient reduction of PPG of more than 20%. The 1-year shunt dysfunction rate was 5.9% vs. 8.1% and 2-year shunt dysfunction rate was 18% vs. 24.6% (NASH group vs. HBV group).

**Table 2:** Portal hemodynamics of patients before and after the treatment.

	NASH (*n* = 38)	HBV (*n* = 82)	*p* value
PP before TIPS (mmHg)	26.3 ± 6.1	30.1 ± 4.7	<0.001
WHVP before TIPS (mmHg)	24.1 ± 5.3	27.6 ± 5.5	0.001
FHVP before TIPS (mmHg)	8.8 ± 3.0	9.7 ± 3.4	0.186
HVPG before TIPS (mmHg)	15.3 ± 3.8	18.0 ± 4.8	0.003
PPG before TIPS (mmHg)	18.0 ± 3.7	20.0 ± 3.4	0.005
PPG after TIPS (mmHg)	7.6 ± 4.1	9.2 ± 3.8	0.040
Portal vein velocity after TIPS (cm/s)	38.6 ± 16.3	38.8 ± 12.7	0.756

PP, portal pressure; WHVP, wedged hepatic venous pressure; FHVP, free hepatic venous pressure; PPG, portosystem pressure gradient; HVPG, hepatic venous pressure gradient; TIPS, transjugular intrahepatic portosystemic shunt.

### Rebleeding

**Figure 1:**
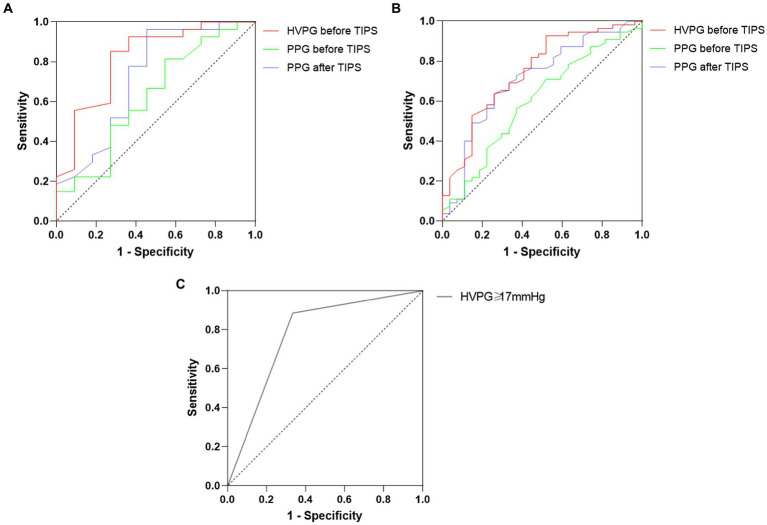
**(A)** ROC curve for the value of HVPG and PPG before and after TIPS in predicting variceal rebleeding in patients with NASH cirrhosis. **(B)** ROC curve for the value of HVPG and PPG before and after TIPS in predicting variceal rebleeding in patients with HBV cirrhosis. **(C)** ROC curve for the value of HVPG ≥17.0 mmHg after TIPS in predicting variceal rebleeding in patients with NASH cirrhosis.

**Figure 2:**
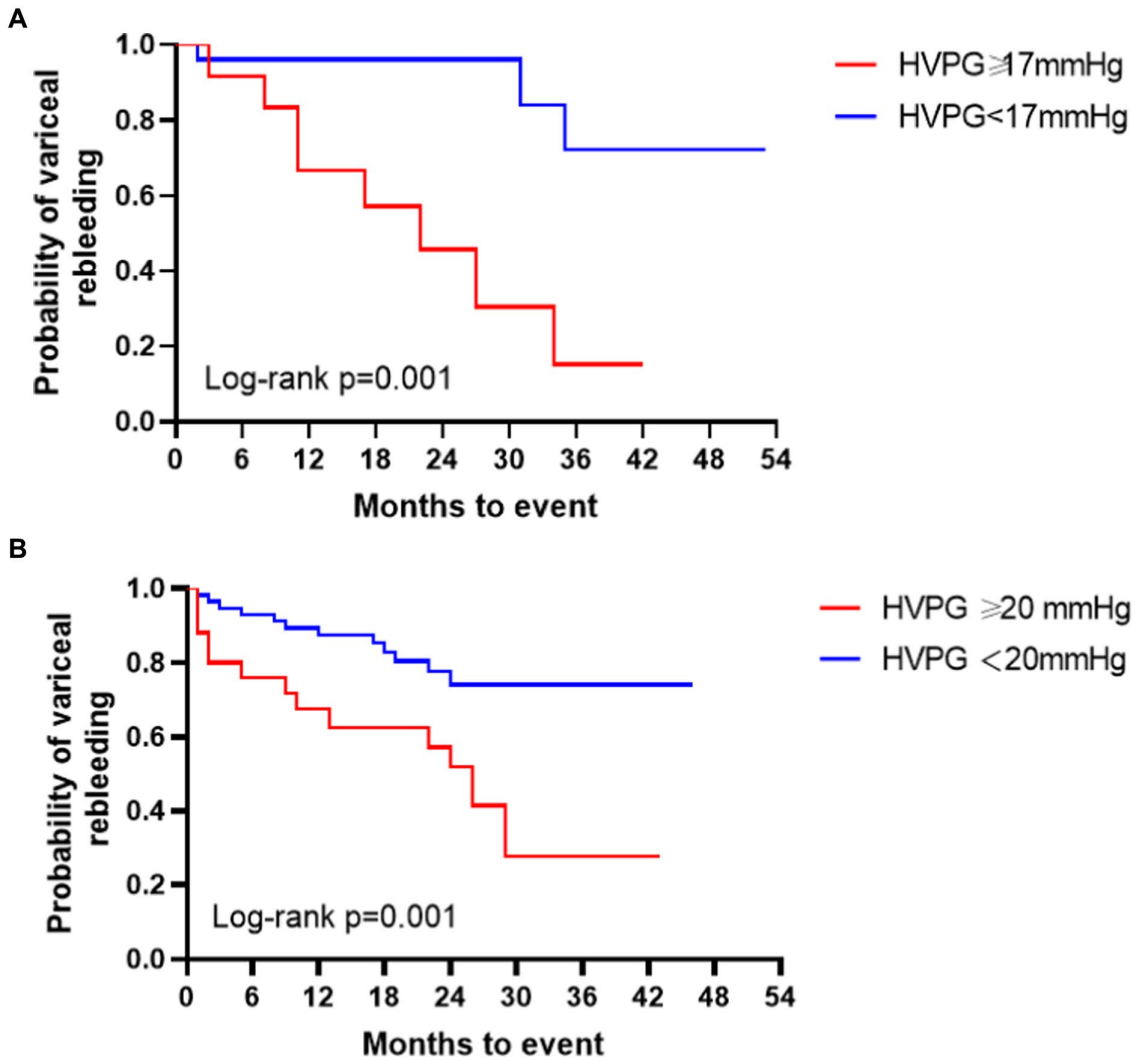
**(A)** Survival curves of the probability of variceal rebleeding in patients with NASH cirrhosis based on HVPG level. **(B)** Survival curves of the probability of variceal rebleeding in patients with HBV cirrhosis based on HVPG level.

During the follow-up, a total of 38 patients (11 NASH patients and 27 HBV patients) had at least one rebleeding. Analyzed by competing risk analysis, there were no significant differences in the cumulative incidence of rebleeding at 6 months (5.3% vs. 12.2%), 1 year (14.6% vs. 18.6%) or 2 years (22.7% vs. 32.9%) between the two groups. Furthermore, Kaplan–Meier survival curves indicated no significant difference in the overall rebleeding rate between NASH and HBV cirrhosis. Variceal rebleeding patients had higher baseline HVPG and PPG levels than nonrebleeding patients in both groups. Patients with PPG greater than 12 mmHg after TIPS placement were at higher risk for rebleeding than those without (71.2% vs. 50.0%, *p* = 0.059). Patients with a higher HVPG level of ≥20 mmHg had a significantly higher variceal rebleeding rate than those with an HVPG of <20 mmHg (64.3% vs. 21.7%, *p* < 0.001). According to competing risk model, the observed cumulative probability of variceal rebleeding was significantly higher in those with an HVPG ≥20 mmHg than in those with an HVPG <20 mmHg at the 6-month (25.0% vs. 5.4%), 1-year (36.2% vs. 11.4%) and 2-year (49.9% vs. 23.0%) follow-ups. In logistic regression, HVPG ≥20 mmHg was associated with an increased risk of variceal rebleeding (HR 6.48; 95% CI 2.59–16.23; *p* < 0.001) compared with an HVPG <20 mmHg. The effect with the PPG after TIPS did not reach significance. The c-statistic for baseline HVPG and PPG for predicting variceal rebleeding were 0.82 (95% CI 0.66–0.97; *p* = 0.002) and 0.72 (95% CI 0.53–0.92; *p* = 0.027) in NASH patients, and the optimal threshold for baseline HVPG and PPG were ≥ 17.0 mmHg (specificity 85.2%, sensitivity 72.7%) and ≥ 20.9 mmHg [specificity 96.3%, sensitivity 54.5% (Figure 1A)]. The ROC curve of HVPG greater than 17 mmHg was shown in Figure 1C. In the HBV group, the c-statistic for baseline HVPG and PPG for predicting variceal rebleeding were 0.75 (95% CI 0.36–0.86; *p* < 0.001) and 0.71 (95% CI 0.59–0.83; *p* = 0.002), and the optimal threshold for baseline HVPG and PPG were ≥ 21.6 mmHg (specificity 48.1%, sensitivity 92.7%) and ≥ 20.2 mmHg [specificity 74.1%, sensitivity 63.6% (Figure 1B)]. The ROC cruves of PPG after TIPS placement were not statistically different. Elevation of the baseline HVPG level per 1 mmHg increased the rebleeding risk by 1.50 in NASH cirrhosis (95% CI 1.11–2.03; *p* = 0.008) and 1.23 in HBV cirrhosis (95% CI 1.09–1.40; *p* = 0.001). The survival curves of variceal rebleeding in the NASH and HBV groups according to HVPG are depicted in Figure 2. The incidence of rebleeding was significantly higher in patients with an HVPG ≥17 mmHg in the NASH group (HR 7.06; 95% CI 1.88–26.56; *p* = 0.001). Multivariate analysis showed that HVPG ≥17 mmHg (HR 9.40; 95% CI 1.85–47.70; *p =* 0.007), lower albumin (HR 1.25; 95% CI 1.06–1.48; *p* = 0.007), and higher GGT (HR 1.02; 95% CI 1.01–1.03; *p* = 0.002) were independent predictors of variceal rebleeding in the NASH cirrhosis group (Table 3). The PPG before and after TIPS had no significance in the multivariate Cox regression model. The median survival time was 22 months for NASH patients with an HVPG ≥17 mmHg and 26 months for HBV patients with an HVPG ≥17 mmHg, although no significant difference was found between the two groups.

**Table 3:** Univariate and multivariate analysis for predictors of variceal rebleeding in NASH cirrhosis.

	Univariate analysis		Multivariate analysis	
Variable	HR (95% CI)	*p* value	HR (95% CI)	*p* value
Overweight/Obese	3.73 (0.98–14.28)	0.054		
HVPG ≥17 mmHg	7.57 (1.96–29.23)	0.003	9.40 (1.85–47.70)	0.007
PPG	1.20 (1.04–1.38)	0.014		
Albumin	0.88 (0.78–1.00)	0.055	0.80 (0.68–0.94)	0.007
GGT	1.01 (1.00–1.02)	0.014	1.02 (1.01–1.03)	0.002
AP	1.01 (1.00–1.02)	0.005		

CI, Confidence interval; HR, Hazard ratio; HVPG, hepatic venous pressure gradient; PPG, portosystem pressure gradient; GGT, gamma-glutamyl transpeptidase; AP: alkaline phosphatase.

### Other complications of cirrhosis

At enrollment, 19 (50.0%) NASH patients had ascites, 3 (7.9%) had encephalopathy, and 2 (5.3%) had acute-on-chronic liver failure. During a mean follow-up of 27.6 months, 13 (34.2%) patients with NASH cirrhosis developed cirrhotic complications other than variceal bleeding, including encephalopathy (7 patients), HCC (1 patient), acute-on-chronic liver failure (7 patients), and ascites (4 patients). Fifty (61.0%) HBV cirrhosis patients had ascites, 10 (12.2%) had encephalopathy, 7 (8.5%) had liver failure, and 4 (4.9%) had HCC at the time of the first visit of the study. Compared with patients with NASH cirrhosis, 42 (51.2%) patients with HBV cirrhosis developed cirrhotic complications other than variceal bleeding (22 patients with encephalopathy, 10 patients with HCC, 7 patients with liver failure, 8 patients with ascites) during 2 years of follow-up. None of patient received liver transplants. Although the NASH group showed a lower total incidence of cirrhotic complication outcomes, the occurrence rates of hepatic encephalopathy (26.3% vs. 39%), acute-on-chronic liver failure (23.7% vs. 34.1%), and ascites (60.5% vs. 70.7%) were similar between two groups. According to the Kaplan–Meier analysis, the incidence of HCC was significantly higher in HBV cirrhosis (17.1% vs. 2.6%, *p* = 0.008). In the NASH group, the HVPG level was significantly higher in patients with cirrhotic complications than in those without complications (16.2 ± 3.9 vs. 13.4 ± 2.8; *p* = 0.026). The prevalence of cirrhotic complications increased with the HVPG level. Each 1 mmHg elevation in HVPG was associated with a 27.8% increase in the risk of clinical events (*p* = 0.035). The c-statistic of HVPG for the predictive value of cirrhotic complications was 0.75 in NASH patients (95% CI 0.59–0.90; *p* = 0.014).

During follow-up, 1 patient with NASH died due to liver failure, and 2 patients with HBV died due to HCC and lethal variceal bleeding. Furthermore, there were no differences in survival between the groups.

## Discussion

HVPG measurement is a reliable method to assess portal hypertension. Nevertheless, the correlation between HVPG and cirrhotic decompensation has not yet been well documented in NASH cirrhosis. HVPG has been verified to contribute to the progression of cirrhotic decompensation in other etiologies. It is suggested that the threshold value of HVPG for risk stratification is likely to be different in NASH cirrhosis (7, 12). In this study, we evaluated the correlation between HVPG levels and cirrhotic complications in NASH cirrhosis.

Our results showed lower PP (26.3 ± 6.1 vs. 30.1 ± 4.7; *p* < 0.001), lower WHVP (24.1 ± 5.3 vs. 27.6 ± 5.5; *p* = 0.001), lower HVPG (15.3 ± 3.8 vs. 18.0 ± 4.8; *p* = 0.003) and lower PPG (18.0 ± 3.7 vs. 20.0 ± 3.4; *p* = 0.005) in NASH cirrhosis with variceal hemorrhage than in HBV cirrhosis. Futhermore, this study confirmed a high agreement between HVPG and PPG in NASH cirrhosis. In the current study, lower HVPG and lower WHVP were found in NASH disease than in HCV disease, and decreases in pressure measurements were observed in different stages of fibrosis, particularly in the lower stage of fibrosis (stage ≤3) (15). Compared with the other etiologies of cirrhosis, these decreased pressure variables of NASH cirrhosis were identical to those in a previous study regarding a similar degree of liver dysfunction (20, 21). Our study indicated that low HVPG levels may lead to variceal bleeding in NASH cirrhosis. The low level of portal pressure in NAFLD has recently attracted much attention, raising the question of whether HVPG measurements may probably be underestimated in NASH cirrhosis. HVPG is is an indirect measure of portal pressure, and its accuracy may be questioned for pre-sinus or post-sinus portal hypertension. Previous studies hypothesized that the potential special vasoreactivity mechanism in NAFLD reduces the effect of fibrosis on portal pressure (12). NASH pathogenesis is correlated with lobular inflammation and portal fibrosis. Portal inflammatory infiltrate leads to a ductular reaction, resulting in progressive fibrosis and thus an increase in portal vascular resistance (22, 23). It has also been postulated that increased perisinusoidal pressure caused by biliary injury may influence the accuracy of portal pressure in NASH (24, 25). Moreover, these studies have raised concerns about whether portal hypertension in NASH can be perfectly distinguished by HVPG measurement. Decreased HVPG values for staging fibrosis in NASH have been verified. The measurement of HVPG in NASH and HCV etiology shared the same and strong correlation with the stage of fibrosis (15). HVPG has been clinically significant in the prognosis of cirrhotic complications in compensated and decompensated cirrhosis in NASH cirrhosis, as in other etiologies (14, 26).

In this study, the results suggested that the measurement of HVPG was an accurate predictor of portal hypertension in NASH cirrhosis, such as recurrent variceal bleeding. Although the optimal baseline HVPG threshold for predicting rebleeding of NASH cirrhosis was lower than 20 mmHg, univariate and multivariate analyzes revealed that HVPG ≥17 mmHg was an independent predictor for variceal rebleeding. The median survival time was shorter in NASH patients than in HBV patients when the HVPG was greater than 17 mmHg, although no survival difference was observed. Likewise, our study showed a strong correlation between rebleeding episodes and HVPG elevation. The relationship between high HVPG and complications in NASH cirrhosis has been demonstrated in our study, which can help us corroborate the specific predictive value of HVPG for predicting the development of variceal bleeding. Previous reports on portal hemodynamics indicated that HVPG of ≥20 mmHg had been shown to significantly correlate with a high incidence of cirrhotic complications (27–29). As mentioned above, the role of the HVPG in predicting the occurrence of cirrhotic complications in decompensated NASH cirrhosis is controversial. It is difficult to identify NASH patients at high risk for liver-related complications, especially those with an HVPG <10 mmHg (14). The predictive factors for cirrhotic complications have received much attention in NASH cirrhosis, and identifying independent predictors for portal hypertensive complications is important for patients with NASH cirrhosis (30). In a study of a large cohort with 475 patients with biopsy-proven NASH from the simtuzumab trials, higher HVPG, both at baseline levels and elevated levels over time, was associated with a high risk of cirrhosis-related clinical events (14). With every 1 mmHg increase in HVPG, the associated risk of decompensation events increased by 15%. We also noticed a difference in the risk estimates of decompensation between our study and a previous study. Perhaps because this study only involves patients with decompensated cirrhosis, the risk of variceal bleeding is likely to be exaggerated. Our findings on the prognostic value of HVPG for risk stratification in NASH cirrhosis patients are consistent with those of Sanyal et al. (14), which showed the high prognostic value of HVPG for predicting cirrhotic decompensations and survival.

Few studies have focused on evaluating the predictive value of HVPG for rebleeding risk in NASH cirrhosis patients with variceal bleeding. According to our results, the accuracy of HVPG for predicting variceal rebleeding in NASH cirrhosis is superior to that in HBV cirrhosis. Our observation verifies a significant correlation between increased HVPG and high rebleeding risk. High portal pressure and high variceal pressure are recognized causes of variceal bleeding; hence, HVPG is a well-known useful predictor for variceal bleeding in cirrhotic patients. HVPG ≥20 mmHg correlates with grades of varices and increased risk of continued and recurrent variceal bleeding, which has been shown in many experimental and clinical studies (10, 28, 31, 32). Our findings suggest that an HVPG ≥17 mmHg is a valuable predictor for evaluating the risk of complications in patients with NASH cirrhosis. Thus, early evaluation of HVPG provides an opportunity for early intervention in these at-risk patients.

The limitation of this study is the relatively small sample size. Another limitation is that the HBV group is not comparable to the NASH group with respect to baseline characteristics, which might be inadequate to accurately describe the predictive value of HVPG in NASH cirrhosis. NASH cirrhosis has been projected to exceed virus cirrhosis and become the leading cause of cirrhosis worldwide (33). The number of NASH patients with cirrhosis is still limited due to slow disease progression, which impedes the assessment of long-term survival. Considering that the average follow-up in this study was approximately 2 years, whether HVPG has a good prognostic value for variceal bleeding in NASH cirrhosis awaits further investigation. We fully anticipate that further studies will explore the predictive value of HVPG for other complications of advanced cirrhosis due to NASH, including liver failure, hepatic encephalopathy, hepatorenal syndrome and hepatopulmonary syndrome. The hemodynamic measurement of portal pressure is invasive and relatively expensive, which limits its large-scale application. In that instance, a new minimally invasive and cost-effective method is expected to be a replacement for HVPG, showing a favorable prognostic value for long-term outcomes in NASH patients.

In conclusion, this study showed that patients with NASH cirrhosis had lower HVPG valus and lower PPG values and similar prevalence of cirrhosis-related complications after acute variceal bleeding compared with HBV cirrhosis. NASH cirrhotic patients with low HVPG values may present with variceal bleeding. According to the predictive value for variceal rebleeding, HVPG is a feasible accurate and valuable means of risk assessment in NASH cirrhosis. The presence of high HVPG contributes to stratifying high-risk patients and leads us to a deeper understanding of the management of NASH patients. Considering the rising trend in the prevalence of NAFLD, regular clinical evaluation and monitoring of liver-related events are recommended in these patients with high HVPG. Accordingly, stratification based on HVPG level is a promising risk stratification among patients with cirrhosis, especially in NASH cirrhosis.

A preprint has previously been published (34).

## Data availability statement

The raw data supporting the conclusions of this article will be made available by the authors, without undue reservation.

## Ethics statement

The studies involving human participants were reviewed and approved by the Ethics Committee of the Second Affiliated Hospital of Chongqing Medical University. The patients/participants provided their written informed consent to participate in this study.

## Author contributions

YS and BN conceived and wrote the manuscript. YS, XW, and GX participated in data acquisition and statistical analysis. WS and BN revised and edited the manuscript and supervised the study. All authors contributed to the article and approved the submitted version.

## Funding

This study was supported by the medical study plan of Chongqing Health Commission, Grant 2016HBRC003.

## Conflict of interest

The authors declare that the research was conducted in the absence of any commercial or financial relationships that could be construed as a potential conflict of interest.

## Publisher’s note

All claims expressed in this article are solely those of the authors and do not necessarily represent those of their affiliated organizations, or those of the publisher, the editors and the reviewers. Any product that may be evaluated in this article, or claim that may be made by its manufacturer, is not guaranteed or endorsed by the publisher.

## Abbreviations

HVPG: hepatic venous pressure gradient
NASH: nonalcoholic steatohepatitis
HCV: hepatitis C virus
HBV: hepatitis B virus
TIPS: transjugular intrahepatic portosystemic shunt
PPG: portosystem pressure gradient
BMI: body mass index
HCC: hepatocellular carcinoma
FHVP: free hepatic venous pressure
WHVP: wedged hepatic venous pressure
ALT: alanine aminotransferase
INR: international normalized ratio
GGT: gamma-glutamyltransferase
HR: hazard ratio
CI: confidence interval
CSPH: clinically significant portal hypertension
